# Studies of Evaluation Methods for Resistance to Fusarium Wilt Race 4 (*Fusarium oxysporum* f. sp. *vasinfectum*) in Cotton: Effects of Cultivar, Planting Date, and Inoculum Density on Disease Progression

**DOI:** 10.3389/fpls.2022.900131

**Published:** 2022-06-13

**Authors:** Jinfa Zhang, Abdelraheem Abdelraheem, Yi Zhu, Heather Elkins-Arce, Jane Dever, Derek Whitelock, Kater Hake, Tom Wedegaertner, Terry A. Wheeler

**Affiliations:** ^1^Department of Plant and Environmental Sciences, New Mexico State University, Las Cruces, NM, United States; ^2^Texas A&M AgriLife Research, El Paso, TX, United States; ^3^Texas A&M AgriLife Research, Lubbock, TX, United States; ^4^Southwestern Cotton Ginning Research Laboratory, Mesilla Park, NM, United States; ^5^Cotton Incorporated, Cary, NC, United States

**Keywords:** Fusarium wilt, race 4, planting date, inoculum density, cultivar, disease progression

## Abstract

Fusarium wilt caused by *Fusarium oxysporum* f. sp. *vasinfectum* race 4 (FOV4) is an early season disease causing root rot, seedling wilt, and death. To develop an appropriate field evaluation method for resistance to FOV4 in cotton breeding, the objectives of this study were to investigate the effects of cultivar, planting date, and inoculum density on disease progression in 2020–2021. Results showed that the usual local mid-April planting had the lowest disease severity (DSR) or mortality rate (MR) in 2020 and 2021. DSR or MR increased at the late April and early May plantings in both years and reached the highest at the early May planting in 2020, while MR in 2021 was followed by a decrease in the late May planting and reached the highest in the mid-June planting. Local daily low temperatures between mid-April and mid-June were favorable for FOV4 infections, whereas daily high temperatures at 35°C or higher suppressed wilt severity. When seedlings at the 2-true leaf stage were inoculated with 10^4^, 10^5^, 10^6^, and 10^7^ conidia ml^−1^ per plant in 2020, DSR was low but a linear relationship between inoculum density and DSR was observed. When a FOV4-infested soil supplemented with artificial inoculation was used, disease progression in three moderately susceptible or moderately resistant cultivars followed a linear model, while it followed a quadratic model in the highly susceptible Pima S-7 cultivar only. Among the other three cultivars, FM 2334GLT had the lowest DSR or MR except for one planting date in both years, followed by PHY 725 RF and Pima PHY 881 RF in ascending order, which were consistent with the difference in regression coefficients of the linear models. This study demonstrates that disease progression curves due to FOV4 can be used to compare responses to FOV4 infections among cotton genotypes in cotton breeding and genetic studies, regardless of planting date and inoculation method.

## Introduction

Cotton (*Gossypium* spp.) is the most important fiber crop for the textile industry and an important oilseed crop globally. Cotton is grown in 17 southern states of the United States, with the highest production in Texas [40% of U.S. cotton production in recent years (USDA Economic Research Service, 2022)]. Fusarium wilt in cotton is caused by the soil- and infrequently seed-borne fungal pathogen, *Fusarium oxysporum* f. sp. *vasinfectum* (FOV). Among eight pathogenic races reported worldwide (Hillock, 1992), races 1, 2, 3, 4, and 8 and several new genotypes have been identified in US cotton (Davis et al., 2006; Holmes et al., 2009; Cianchetta and Davis, 2015; Cianchetta et al., 2015; Halpern et al., 2020). While race 1 is a predominant race in the US Cotton Belt, FOV race 4 (FOV4) has become one of the most important threats to cotton production in the west and southwest states of the Cotton Belt. FOV4, first reported in India (Armstrong and Armstrong, 1960), was first identified in California in 2001 (Kim et al., 2005) and recently in two counties (El Paso and Hudspeth) of Texas (Halpern et al., 2018; Bell et al., 2019; Davis et al., 2022) and one county (Dona Ana) of New Mexico (Zhu et al., 2019, 2020, 2021b; Zhang et al., 2020b). Different genotypic frequencies of FOV4 have been identified among California, Texas, and New Mexico isolates (Bell et al., 2019; Diaz et al., 2021; Zhu et al., 2021a; Liu and Wagner, 2022).

Fusarium wilt caused by FOV4 is an early season disease that causes seedling wilt and death immediately after emergence until the late square stage (Zhang et al., 2020c). After the square stage, most infected plants exhibit chlorotic, necrotic, or wilting leaves, although plant deaths are also observed (Zhang et al., 2020c). Therefore, low temperature early in the planting season of the spring may play an important role in FOV4-associated disease development. Greenhouse evaluations of cotton germplasm lines for FOV4 resistance should be conducted under low-temperature conditions (Zhang et al., 2020a). However, the effect of different planting dates on FOV4 disease development in different cotton cultivars has not been studied. In a 3-year field study using four cotton genotypes with three biweekly planting dates in California, Jeffers and Roberts (1993) showed that delay in planting cotton had the important potential to manage Fusarium wilt (caused by likely race 1). However, the exact effect of temperature on reducing FOV infections from the delayed planting was unknown. Based on a study on four planting dates between 16 October and 26 November 2002 in Australia (Allen, 2005), the increase in the end-of-season survival of cotton plants (from FOV unique Australian type) by delay in planting was attributed to the reduced rainfall in the spring, even though soil temperatures were also increased. However, in the arid southwest and west Cotton Belt (California, New Mexico and Texas) of the US where FOV4 is found, rainfall during the planting and early cotton growing season are minimal. Soil moisture is more uniform because when rain is limited, supplementary irrigation is utilized for seedling establishment. Soil wetness differences in this region may have little effect on FOV4 infections, but temperature differences during April and May can be substantial.

Fusarium wilt caused by FOV4 is dependent on not only temperature (Zhang et al., 2021a) but also inoculum density (Hao et al., 2009). In a greenhouse study, Hao et al. (2009) showed that inoculum density at and below 10^2^ conidia g^−1^ of potting soil mix did not cause wilt symptoms and reductions in plant growth as measured by plant weight, height, and the number of nodes. Disease severity increased from inoculum levels of 10^3^ conidia g^−1^ of soil to 10^5^ and 10^6^ for the highly susceptible cultivar Pima DP 744 and the moderately susceptible Upland Ultima, respectively. However, whether the effect of FOV4 inoculum density on disease development is affected by planting dates is currently unknown.

Based on our previous studies in a field with FOV4-infestations in 2018–2021 (Zhang et al., 2020c; Zhu et al., 2021a), mortality rates caused by FOV4 ranged between 80 and 90% in highly susceptible Pima S-7 and Pima DP 744 which were planted between April 24 and May 5. No difference was observed in FOV4-caused wilt severity during this period when temperatures were low (Zhang et al., 2020c; Zhu et al., 2021a). However, it was unknown if the further delay in planting would reduce disease severity on cotton infected with FOV4 and if disease progression differed among cultivars with different levels of resistance among different planting dates. Here, we carried out a series of experiments to investigate the effect of planting date, cotton cultivar, and inoculum density on disease development caused by FOV4. Results also allowed a comparison of disease progressions among four different cultivars, representing the first such study on FOV4 resistance in cotton.

## Materials and Methods

### 2020 Tests

Five planting dates—April 15 (4/15) and 25 (4/25), May 5 (5/5) and 15 (5/15), and June 5 (6/5) with a 10-day interval except for the last planting date with 21-day interval were selected. Three cultivars (Upland FM 2334GLT and PHY 725 RF, and Pima PHY 841 RF) with different responses to FOV4 (Zhu et al., 2021a,b) were used. Ten cotton seeds from each cotton cultivar were planted in a 10-cm pot (as a replication) filled with non-infested potting soil (Miracle-Gro Moisture Control Potting Mix with fertilizers for plant growth up to 6 months, Scott Co., Marysville, OH, USA). Inoculum density of 10^4^, 10^5^, 10^6^, and 10^7^ conidia ml^−1^ from a local virulent FOV4 isolate (Zhang et al., 2020a; Zhu et al., 2021b) were used to inoculate seedlings at the 2-true leaf stage by pouring 25 ml of each inoculum treatment to the soil surface without root wounding, followed by a light irrigation. For each inoculum density, a randomized complete block design with three replications was performed. To mimic the natural field conditions, the entire experiment was conducted twice the same time outside of the cotton greenhouse, Fabian Garcia Plant Science Center, New Mexico State University, Las Cruces, NM, USA. Plants were watered daily, and no additional fertilizer was applied.

Foliar disease severity ratings (DSR) were measured at 30 days post-inoculation (dpi). Each plant (with ~10 plants per replication in a pot) was rated based on a 0–5 rating scale with 0 for no symptom and 5 for plant death (Sanogo and Zhang, 2016; Zhang et al., 2020a), adopted from Zhang et al. (2012). Disease incidence (DI, percentage of symptomatic plants with ratings 1–5) and average DSR for each cultivar in each replication was calculated.

### 2021 Tests

In 2021, four planting dates [April 16 (4/16, day 0), May 7 (5/7, day 21) and 26 (5/26, day 40), and June 16 (6/16, day 62)] were chosen. For each planting date, a pre-infested soil (10^4^ conidia g^−1^ of soil from the same local virulent FOV4 isolate used in 2020) was used to mimic infested field conditions and to also increase the wilt incidence and severity. The pre-infested soil was from potting soil (the same as used in 2020) previously grown with cotton and inoculated with FOV4 followed the same method used in the 2020 tests. No other soilborne pathogens were observed and isolated. Seeds from two Upland cultivars—FM 2334GLT and PHY 725 RF (the same as used in 2020) and two Pima cultivars—Pima PHY 881 RF (moderately resistant or susceptible) and Pima S-7 (highly susceptible) were planted in 19-L pots where 50 seeds for each cultivar were sown into 25 holes (2 seeds per hole) in each pot. A 4 × 4 Latin square design with a randomized complete block design and four replications was used to arrange each replicated test. At the 1–2 true leaf stage, 2 ml of 1 × 10^6^ conidia ml^−1^ suspension per plant were poured onto the soil surface in each pot, followed by a light irrigation. Daily irrigation and weekly application of fertilizers (due to the reuse of a used pre-infested soil) were made. All the tests were performed at the same location as in 2020. At 15–17 days after planting (DAP), total seedlings (~50 plants per replication in a pot) were counted. At 15–17, 22–23, 30–32, and 36–37 DAP, dead seedlings were counted and removed. Mortality rate (MR) was then calculated as the percentage of dead seedlings in each plot. The proportion of plants that died after emerging (MR = mortality on a 0–1 scale where 1 = 100% survival) was counted on various dates, with a range of 13–60 days after inoculation with FOV4.

### Daily Temperatures

The temperature data including daily low, high, and mean temperature from middle April to early or the end of July in 2020 and early August 2021 were obtained from a local weather station.

### Statistical Analysis

An analysis of variance (ANOVA) was separately performed for the results in 2020 and 2021 using SAS 9.4 (SAS Institute Inc., Cary, NC, USA) based on a general linear model (GLM) procedure to determine the statistical significance of various sources of variation. For 2020, because of the lack of significant interactive effects from genotype and inoculum density with the test (each of the two tests had three replications), the two tests for each planting date were combined into six replications for ANOVA. Because of the significant effects related to the planting date and its interactions with cultivar in both 2020 and 2021, results from individual planting dates were separately analyzed. The least significant difference (LSD) at *p* < 0.05 was used to separate means. Correlation analyses were further performed between average daily low, high, and mean temperature and DSR or MR among planting dates at the same evaluation date using Excel.

The DSR results as a dependent variable were plotted against inoculum density as an independent variable for each of the three tested cultivars using a linear regression model. The rate of mortality over time was examined with linear, monomolecular, and several curvilinear models, including quadratic and exponential models. The acceptable models had slope parameters that were significant at *p* = 0.05, and a random distribution of data points around the predicted line. If there was more than a single acceptable model, then the linear model was used unless another model had a substantially higher *R*^2^ value.

## Results and Analysis

### 2020 Experiments

**Figure 1:**
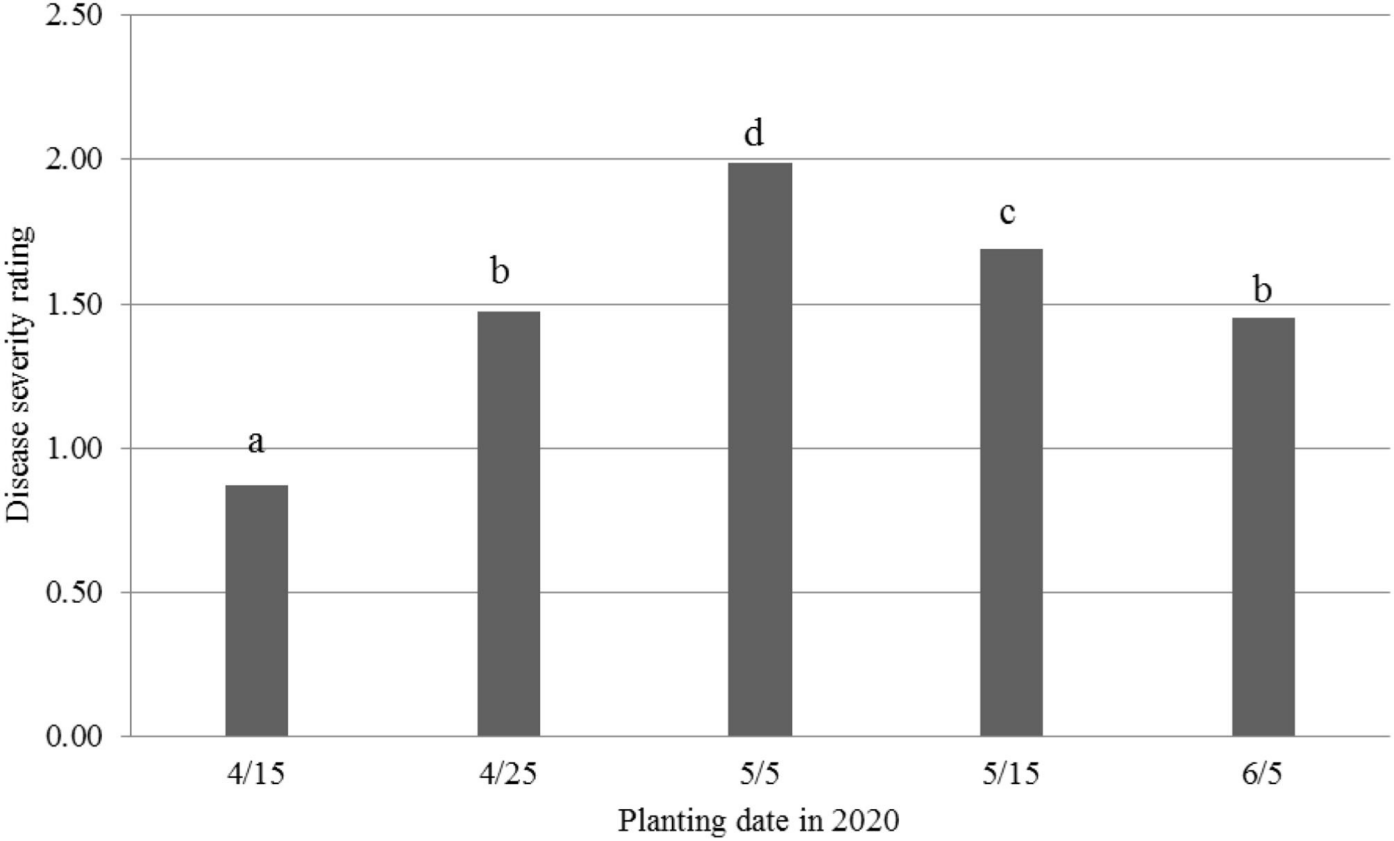
Disease severity ratings from *Fusarium oxysporum* f. sp. *vasinfectum* race 4 in cotton at five different planting dates in 2020, 30 days post-inoculation. Individual plants were rated for disease severity based on the following scale: 0, no symptom; one wilted cotyledon; 2, two wilted cotyledons or two cotyledons abscised; 3, first true leaf wilted or three leaves abscised; 4, whole plant wilted or more than three leaves abscised; and 5, complete defoliation or plant death. Different letters above the bars indicate significant differences.

Mortality was very low (1%−5%) for the five planting dates, i.e., April 15 (4/15) and 25 (4/25), May 5 (5/5) and 15 (5/15), and June 5 (6/5). However, disease incidence was similar to that observed in a greenhouse or temperature-controlled conditions (Zhang et al., 2020a, 2021b; Zhu et al., 2021a). Therefore, DSR was then used for the following analyses. Overall (Figure 1), the 5/5 planting date had the highest DSR (1.99), followed by the 5/15 planting date (1.69). Next were the 4/25 and 6/5 planting dates with similar DSR (1.45–1.48), and the 4/15 planting date had the lowest DSR (0.87). It should be pointed out that under actual field conditions, germinating seeds were exposed to FOV4 infections after planting, causing root rot and plant wilt, and mortality immediately after seedling emergence (see the 2021 results).

In general, inoculum density at 10^7^ spores ml^−1^ caused a similar DSR to that at 10^6^ spores ml^−1^, but its DSR was higher than at 10^4^ and 10^5^ spores ml^−1^ on all the planting dates. Across all the planting dates and three cultivars, DSR increased as FOV4 inoculum density increased, and the relationship was linear (with a coefficient of correlation *r* = 0.9903, *p* < 0.01). Averaged across all the planting dates and cultivars, 10^6^ and 10^7^ spores ml^−1^ had significantly higher DSR (1.62–1.68) than that at 10^4^ spores ml^−1^ (with a DSR of 1.44). Inoculum density at 10^5^ spores ml^−1^ had an intermediate DSR (1.54), but it was not significantly different from other inoculum densities (LSD_0.05_ = 0.18).

**Figure 2:**
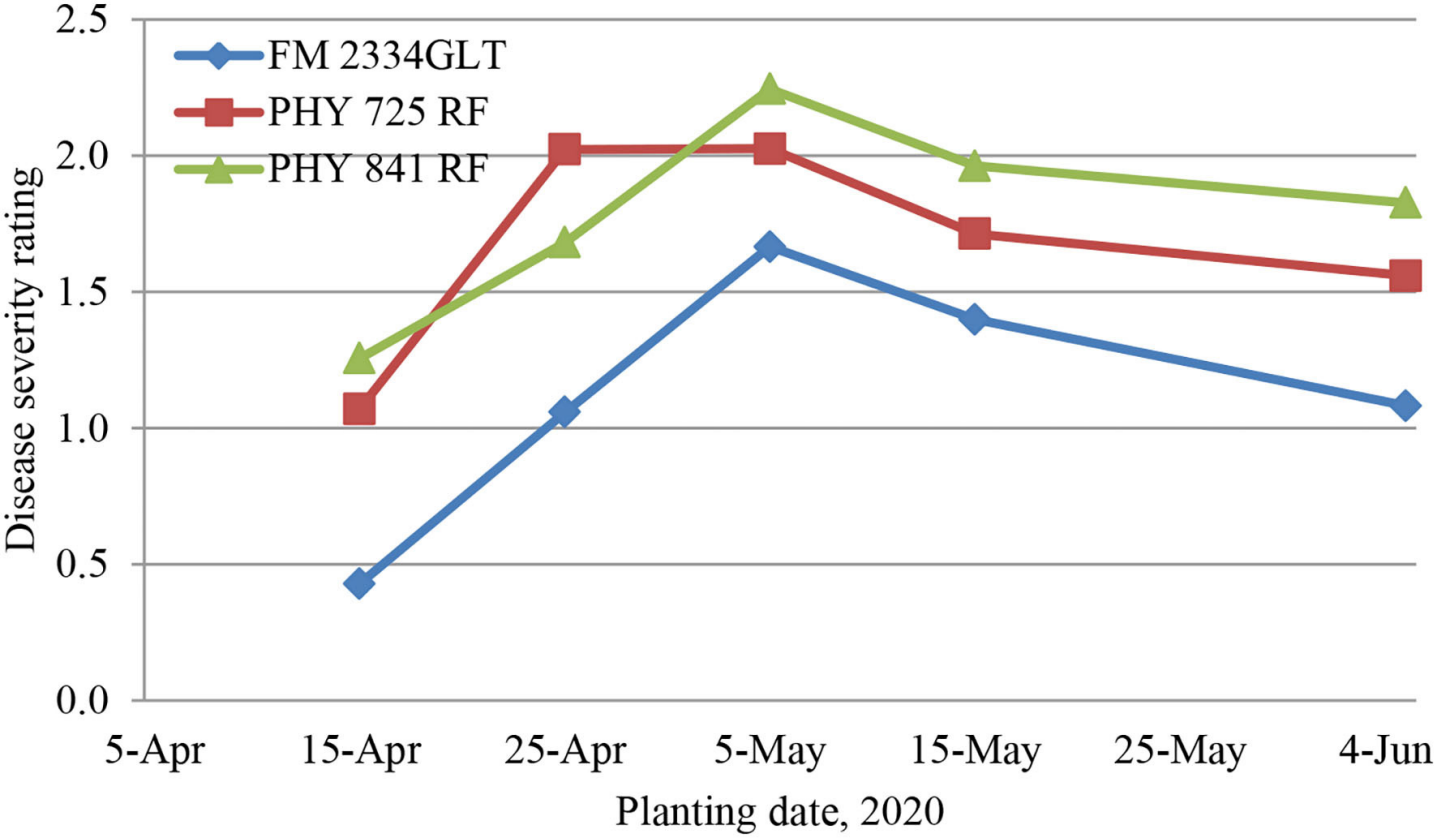
Average disease severity rating in three cotton cultivars from five different planting dates (April 15, April 25, May 5, May 15, and June 5, 2020), 30 days post-artificial inoculation of *Fusarium oxysporum* f. sp. *vasinfectum* race 4. Individual plants were rated for disease severity based on the following scale: 0, no symptom; one wilted cotyledon; 2, two wilted cotyledons or two cotyledons abscised; 3, first true leaf wilted or three leaves abscised; 4, whole plant wilted or more than three leaves abscised; and 5, complete defoliation or plant death.

At all the planting dates, FM 2334GLT had significantly lower DSR than Pima PHY 841 RF and PHY 725 RF (Figure 2). Pima PHY 841 RF had significantly lower DSR at the 4/25 planting date but significantly higher DSR than PHY 725 RF at the two latest planting dates (5/15 and 6/5).

**Figure 3:**
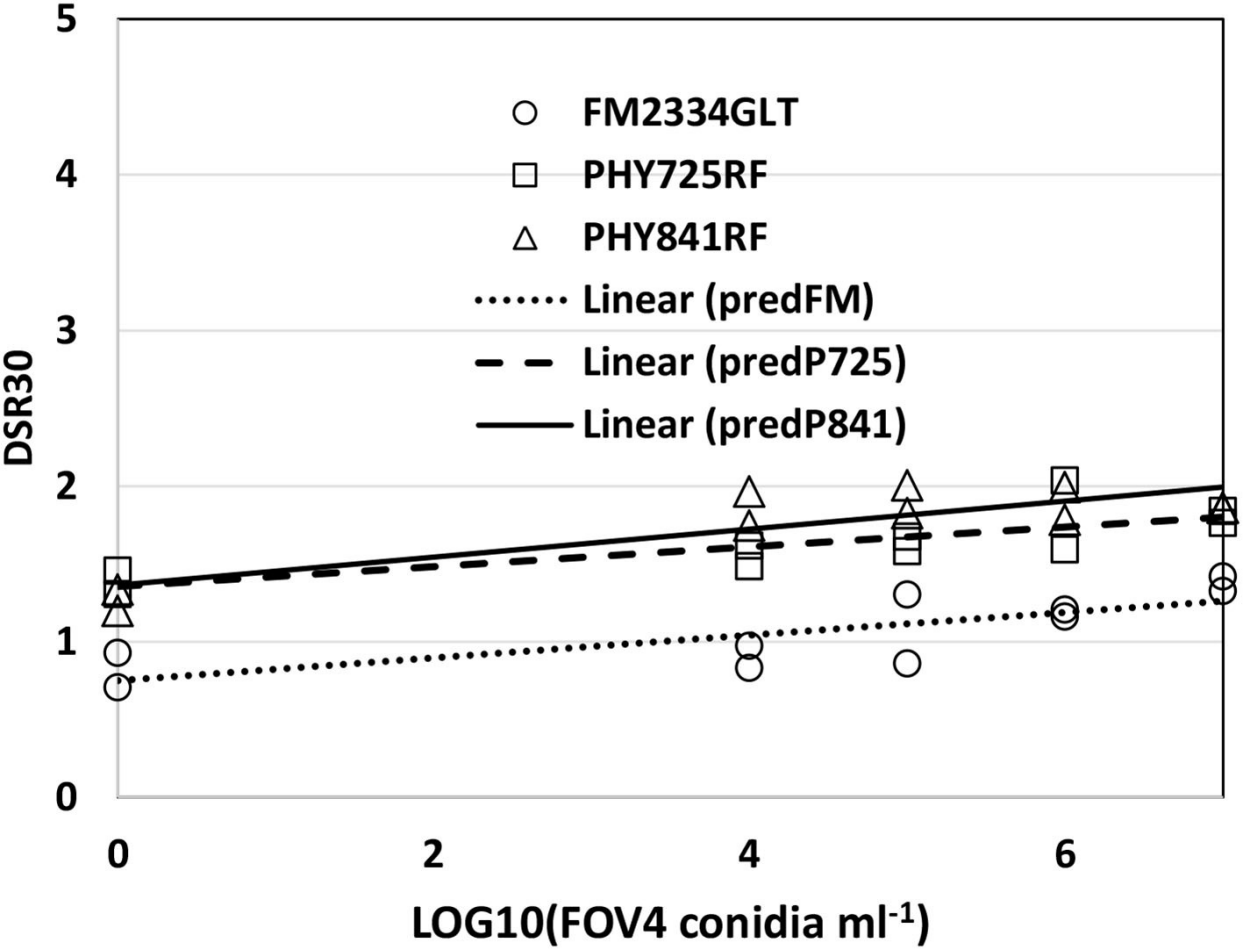
FOV4 symptoms at 30 days after inoculation, as a function of inoculum density in 2020. Plants were rated on a 0–5 scale with 5 being plant death. PredFM, predP725, and P841 are the linear relationship predicted for FM 2334GLT, PHY 725 RF, and PHY 841 RF, respectively.

DSR as a function of inoculation density could be described adequately for all the three cultivars with a linear model (Figure 3) when both tests were combined (because of similar results between tests). The slope values were similar among the three cultivars (FM 2334GLT = 0.0732, PHY 725 RF = 0.0637, and Pima PHY 841 RF = 0.0897), but the intercept term for FM 2334GLT was significantly lower (0.750, with a standard error SE = 0.132) than for PHY 725 RF (1.355, SE = 0.127), and Pima PHY 841 RF (1.365, SE = 0.118). This suggests that early disease symptoms were lower for FM 2334GLT than the other cultivars, but then disease symptoms progressed at a similar rate for all three cultivars.

### 2021 Experiments: Disease Progression by Cultivar at Each Planting Date

**Figure 4:**
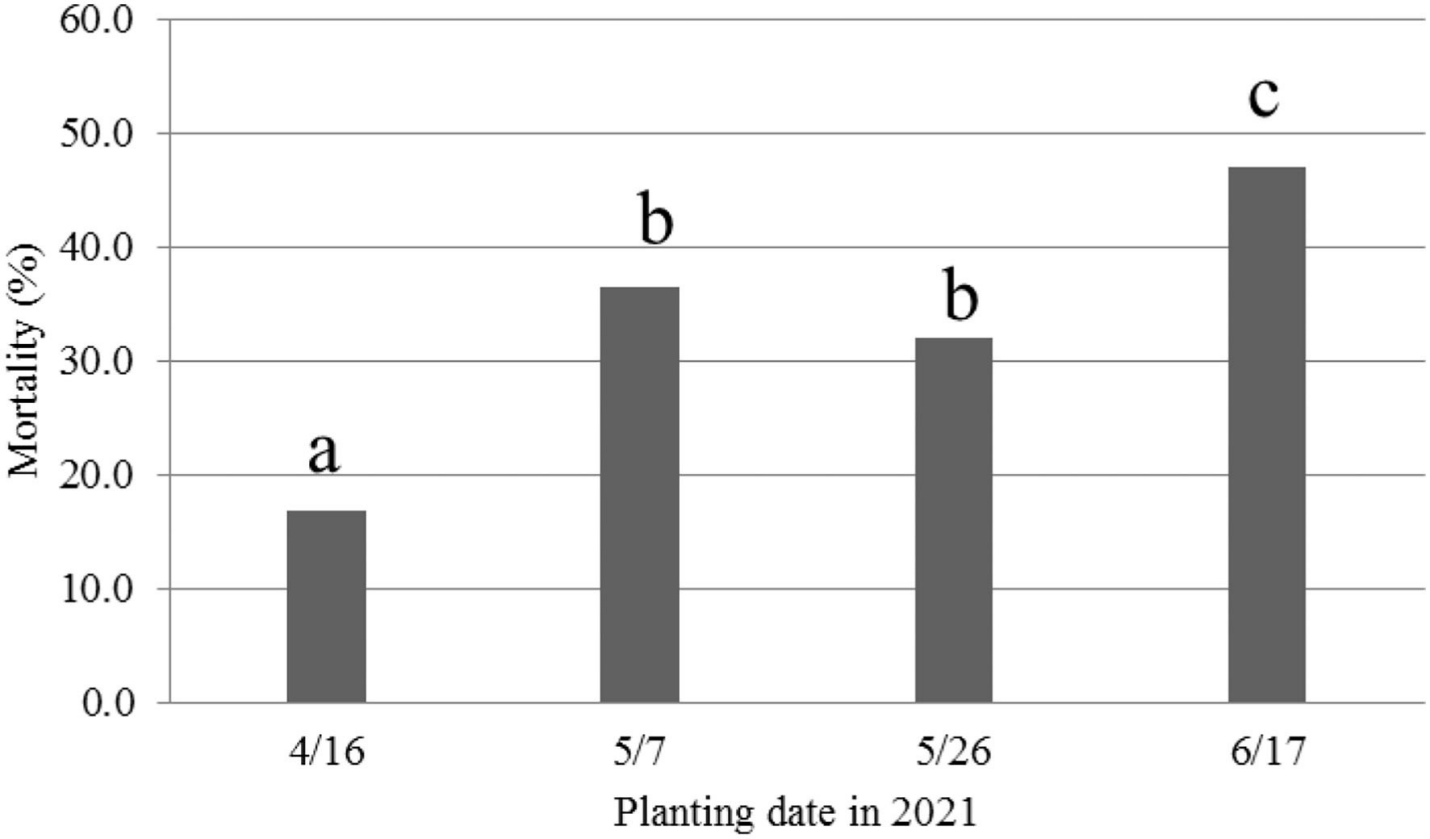
Mortality rate (%) caused by *Fusarium oxysporum* f. sp. *vasinfectum* race 4 at 36 days after planting (DAP) from different planting dates in 2021. Different letters above the bars indicate significant differences.

Because of the use of pre-infested soil in 2021, seedling wilts and deaths were the first symptoms observed immediately after emergence, similar to field conditions. Therefore, MR was used in the following analyses. Overall across the four cultivars, consistent with the 2020 results, the 16 April planting date had the lowest disease severity based on MR, followed by the two May planting dates (Figure 4). However, the 17 June planting had the highest MR, unlike the 2020 study when the 5 June planting date had a significantly lower DSR than the two May planting dates.

**Figure 5:**
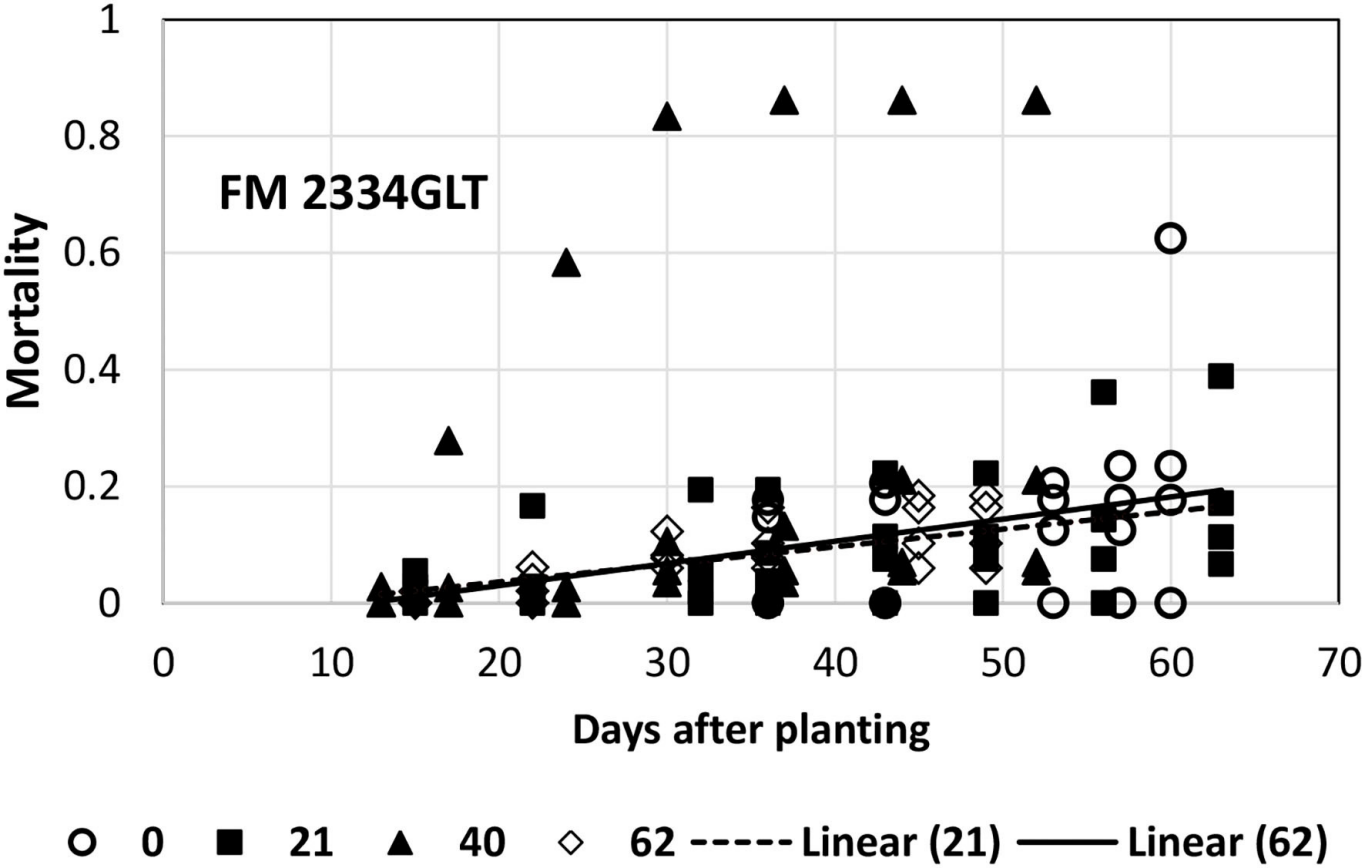
Proportion of plants that emerged and then died (mortality) over time for FM 2334GLT at planting times of day 0, 21, 40, and 62 (day 0 occurred on 16 April) in 2021. Linear models were fitted for day 21 and 62 planting dates.

A linear model in general provided an acceptable fit to the mortality data for FM 2334GLT, PHY 725 RF, and Pima PHY 881RF. Exceptions included FM 2334GLT at 0 (4/16) and 40 (5/26) planting dates. At the 0-day (4/16, earliest) planting date, the data collection was initiated later than the other planting dates, and there was insufficient variability to fit a linear model. The average mortality (slope = 0) provided an adequate description of mortality over time. At the 40-day (5/26) planting date, there was one replicate that performed markedly different than the other three replicates, so that no model could provide a good description of mortality over time (Figure 5). The other two dates (21-day, 5/7; and 62-day, 6/16) could be adequately described by a linear model (Table 1), which had a similar slope and intercept values (Figure 5).

**Table 1:** Model parameters describing mortality (M) as a function of time after planting (T) in 2021.

**Cultivar^**a**^**	**PD^**b**^**	**Model**	**SE (I^**c**^)**	**SE (b_**1**_)**	**SE (b_**2**_)**	**R^**2**^**	***N***
FM	0	None					
FM	21	*M* = −0.024+(0.0030 × *T*)	0.045	0.0011		0.21	32
FM	40	None					
FM	62	*M* = 0.045+(0.0038 × *T*)	0.023	0.00065		0.60	24
P725	0	*M* = −0.125+(0.0054 × *T*)	0.081	0.00161		0.39	20
P725	21	*M* = −0.052+(0.0080 × *T*)	0.023	0.00054		0.88	32
P725	40	*M* = −0.051+(0.0053 × *T*)	0.022	0.00064		0.73	28
P725	62	*M* = 0.070+(0.0092 × *T*)	0.145	0.00416		0.18	24
P881	0	*M* = 0.030+(0.0055 × *T*)	0.091	0.0018		0.34	20
P881	21	*M* = 0.108+(0.0061 × *T*)	0.054	0.0013		0.43	32
P881	40	*M* = −0.055+(0.0059 × *T*)	0.035	0.0010		0.56	28
P881	62	*M* = 0.067+(0.0105 × *T*)	0.142	0.0041		0.23	24
S7	0	*M* = −0.613+(0.0254 × *T*)	0.405	0.0080		0.36	20
S7	21	*M* = 0.134+(0.0266 × *T*)−(0.000245 × *T*^2^)	0.093	0.0052	0.000066	0.73	32
S7	40	*M* = −0.388+(0.0528 × *T*)−(0.000593 × *T*^2^)	0.104	0.0072	0.000111	0.87	28
S7	62	*M* = −0.404+(0.0686 × *T*)−(0.000898 × *T*^2^)	0.185	0.0126	0.000193	0.72	24

^a^*FM = FM 2334GLT; P725 = PHY 725 RF; P881 = PHY 881 RF; S7 = Pima S-7*.

*^b^PD is the planting date. Time 0 was on 16 April, and 21, 40, and 62 represent the number of days after 16 April that each additional set of varieties were planted*.

*^c^I = intercept term for the model. The slope term for the linear model was b_1_ and for the quadratic model were b_1_ and b_2_*.

**Figure 6:**
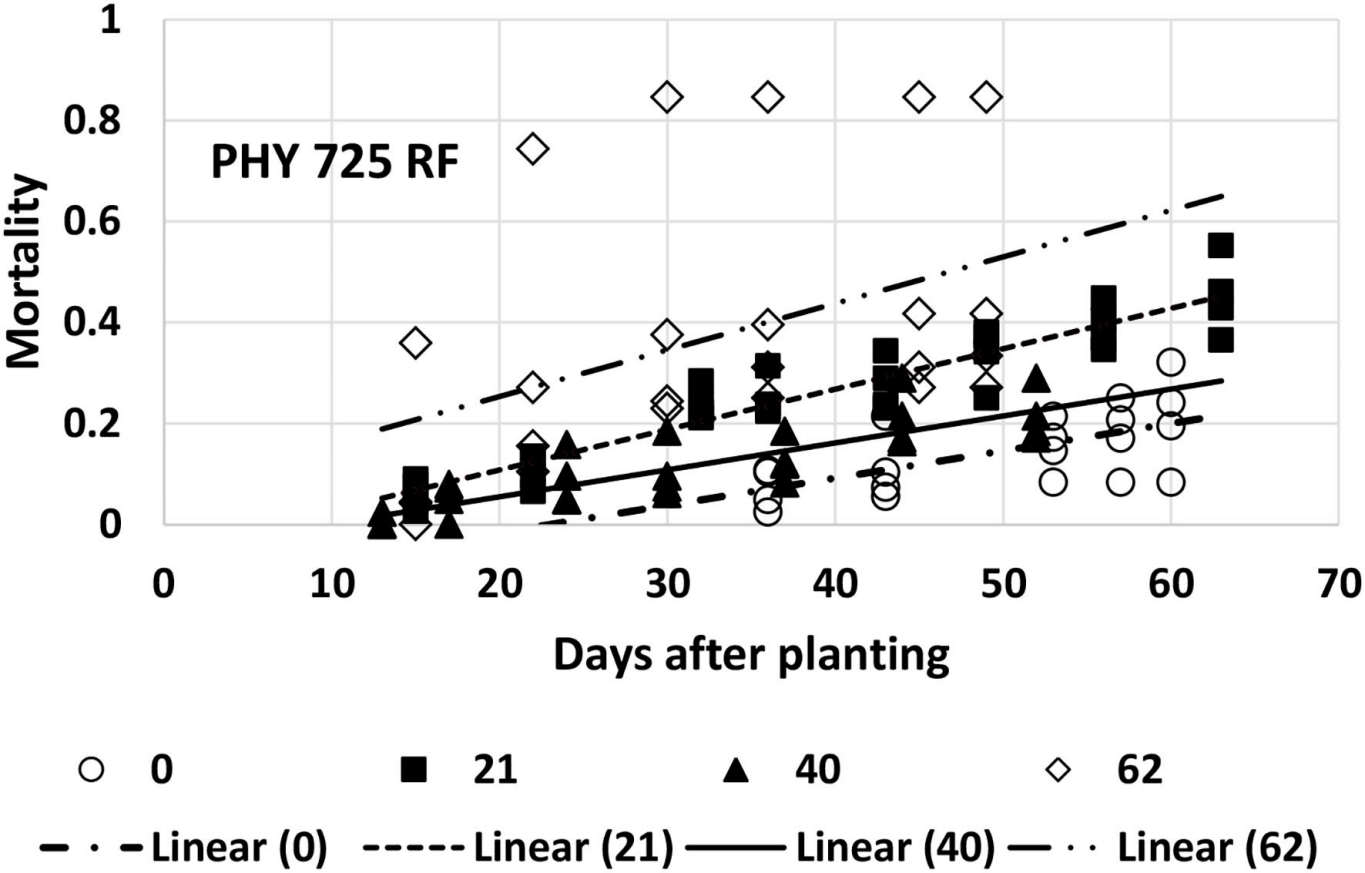
Proportion of plants that emerged and then died (mortality) over time for PHY 725 RF at planting times of day 0, 21, 40, and 62 (day 0 occurred on 16 April) in 2021. Linear models were fitted for all four planting dates (identified as linear 0, 21, 40, or 62).

Mortality for PHY 725 RF could be adequately described with linear models for all four planting dates (Table 1). However, the last planting date had one replicate that had much higher mortality than the other three replications, resulting in a much poorer fit (Table 1, Figure 6), and while the linear model was significant at *p* < 0.04, the 95% confidence interval for the intercept and slope was so wide that it overlapped the values for the other three planting dates. The rate of mortality increase with time was significantly higher for the second planting date (5/7, b_1_ = 0.0080) than for the first (4/16, b_1_ = 0.0054) or third (5/26, b_1_ = 0.0053) planting dates (Figure 6). The intercept values at the second (5/7) and third (5/26) planting dates were similar, and their 95% CI did not overlap the lower intercept value for the first planting date. This suggests that there was more mortality initially for the second and third planting dates than on the first planting date. However, since the data collection was initiated later in the first planting date, the difference could be due to that factor.

**Figure 7:**
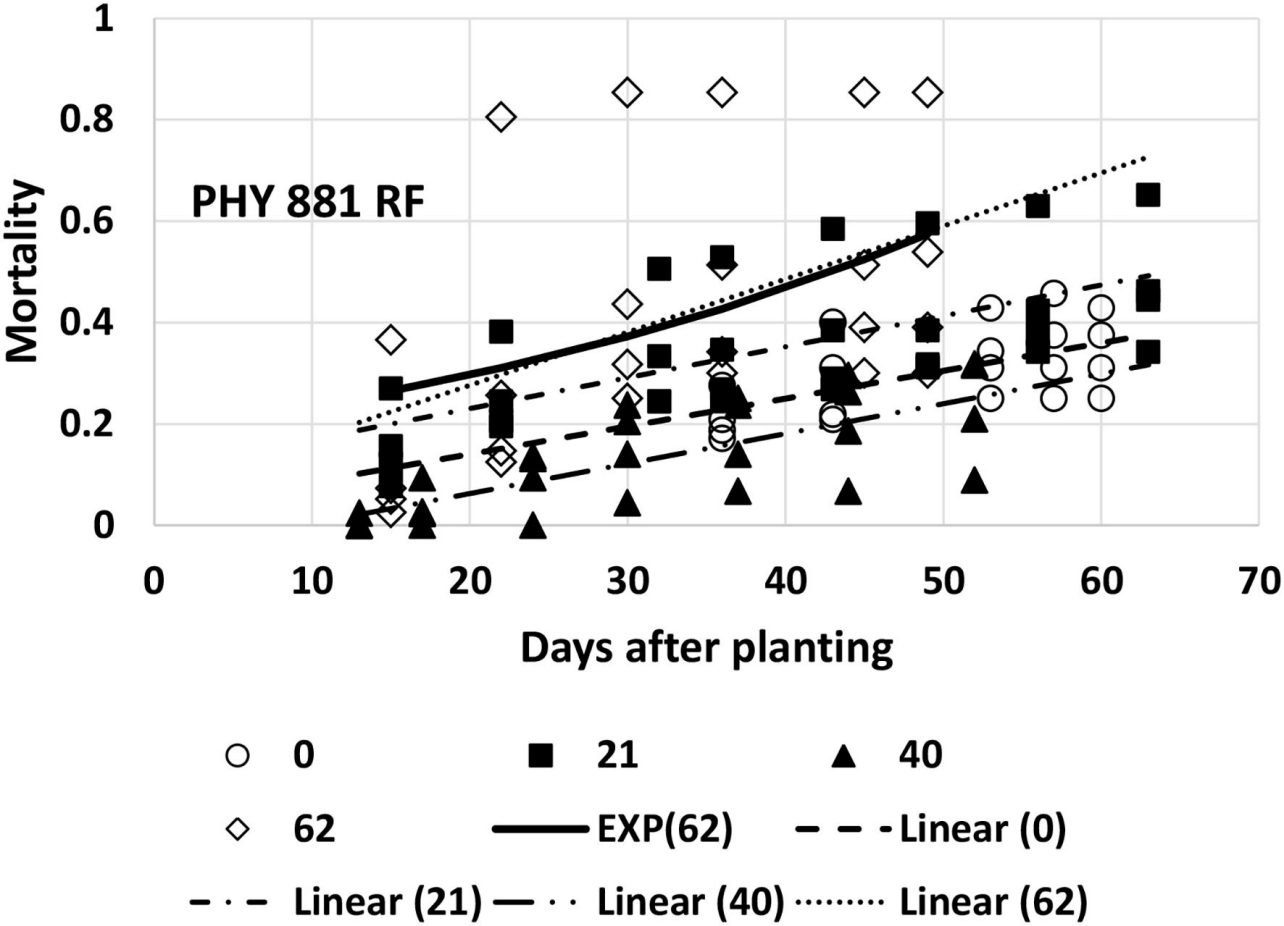
Proportion of plants that emerged and then died (mortality) over time for PHY 881 RF at planting times of day 0, 21, 40, and 62 (day 0 occurred on 16 April). Linear models were fitted for all four dates and are represented as Linear (0, 21, 40, or 62). An exponential model (*Mortality* = *a*×*EXP*^*b*×*T*^), where *a* = 0.188, *b* = 0.0228, and *T* = days after planting was also fitted to the last planting date (EXP62).

Mortality for Pima PHY 881 RF could be adequately described with linear models for all four planting dates. Mortality at the last planting date (day-62, 6/16) was much higher for one replicate than the other three, and the appearance was somewhat curvilinear at this planting time. However, the fit of an exponential equation (both using linearized versions and non-linear modeling) did not provide a significant improvement over the linear model, so the linear model is used. The slope values for the first three planting dates did not differ significantly from each other (Table 1, Figure 7). The 95% CI also did not overlap with the slope from the fourth planting date. However, the large error associated with the slope value for the fourth planting date did cause it to overlap with all other planting dates. The intercept value for the second planting date was significantly higher than for the third planting date (Table 1, Figure 7), suggesting that mortality was initiated earlier for the second planting date than the third planting date.

**Figure 8:**
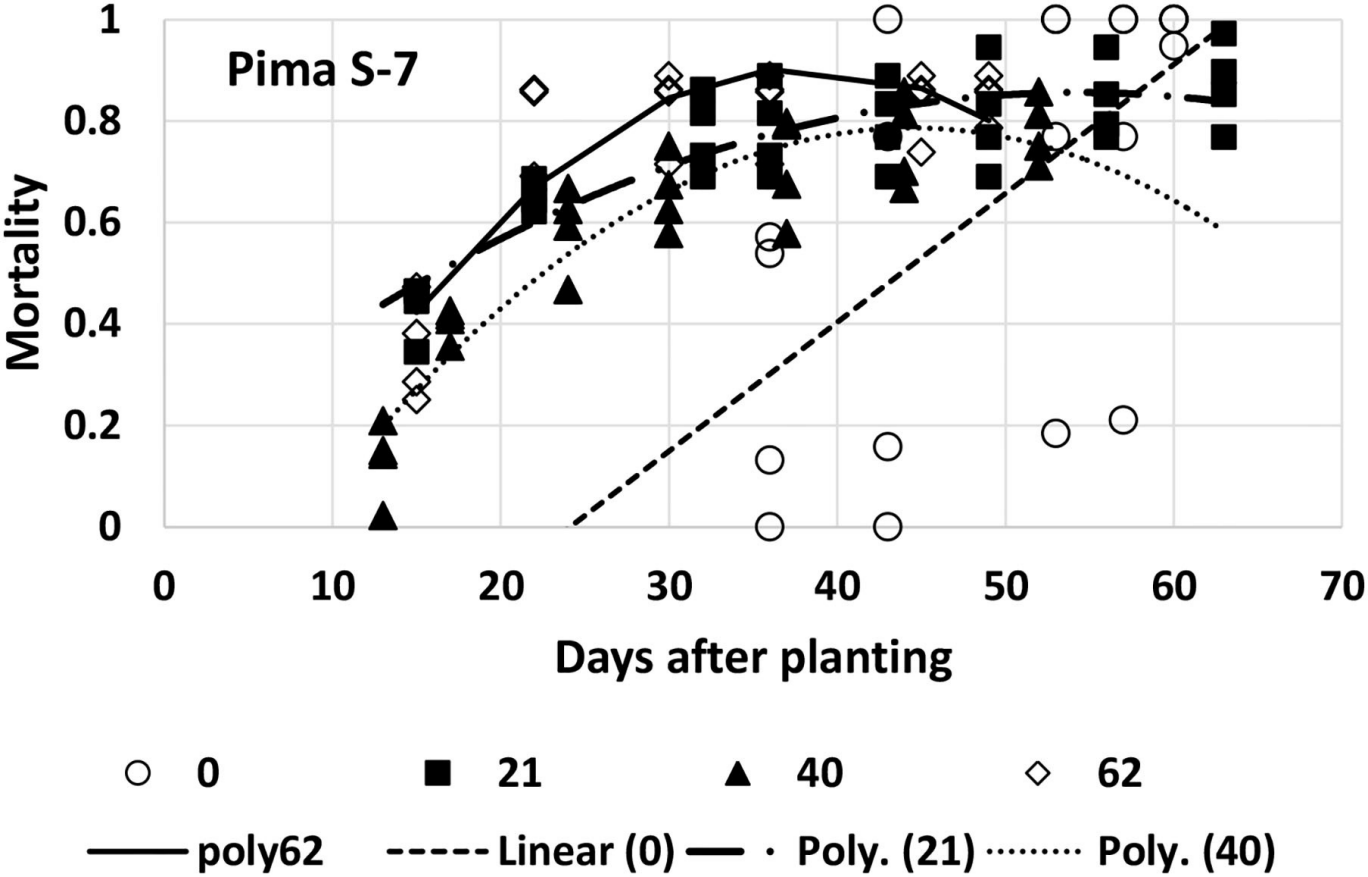
Proportion of plants that emerged and then died (mortality) over time for Pima S-7 at planting times of day 0, 21, 40, and 62 (day 0 occurred on 16 April). A linear model was fitted to day 0 [Linear (0)] and quadratic models for the last three planting dates (Poly 21, 40, 62).

Mortality for Pima S-7 was fitted with a linear model for the first planting date and a quadratic model for each of the other three planting dates (Table 1, Figure 8). When the quadratic model was fitted, it had a much higher *R*^2^ value (average increase of 0.19) than the linear or exponential models. The variability between the replications at the first planting date was high, resulting in a poorly fitting (though significant) linear model. It is difficult to make direct comparisons between parameters with the quadratic models that were fitted to the last three planting dates, but some observations can be made. Plant mortality occurred earlier with Pima S-7 and then slowed down because there were fewer healthy plants left than was seen with the other three varieties. The maximum mortality proportion for the second (5/7), third (5/26), and fourth (6/16) planting dates was predicted to be 0.86 at 54 days after planting, 0.79 at 44 days after planting, and 0.91 at 39 days after planting, respectively. So final mortality was lower at the third planting date than the fourth planting date and also occurred more rapidly at the fourth planting date. The quadratic curve would not have been appropriate to estimate mortality after its maximum value.

At the first planting date (4/26), all the models were at a disadvantage because of the later data collection. Based on the slope values, PHY 725 RF and Pima PHY 881 RF would be considered more resistant than Pima S-7 (Table 1). At the other three planting dates, Pima S-7 cannot be directly compared with the linear models for the other three cultivars, so comparisons will be made only among FM 2334GLT, PHY 725 RF, and Pima PHY 881 RF. At the second planting date (5/7), FM 2334 GLT would be considered more resistant (slower rate parameter) than PHY 725 RF or Pima PHY 881 RF. At the third planting date (5/26), no comparison can be made with FM 2334GLT due to the fact that no model was fit for it, but PHY 725 RF and Pima PHY 881 RF did not differ with respect to the rate of mortality increase over time. At the last planting date (6/16), FM 2334GLT again had a significantly lower rate of mortality than did PHY 725 RF and Pima PHY 881 RF. So, if the earliest planting date is discarded because of late data collection, then a wide range of planting dates (early May to mid-June) can all be effective to characterize mortality rate differences caused by FOV4 between cultivars. The planting date is unlikely by itself to minimize mortality by FOV4, while cultivar resistance/tolerance is effective regardless of planting date.

### Relationship Between Temperature and MR

**Figure 9:**
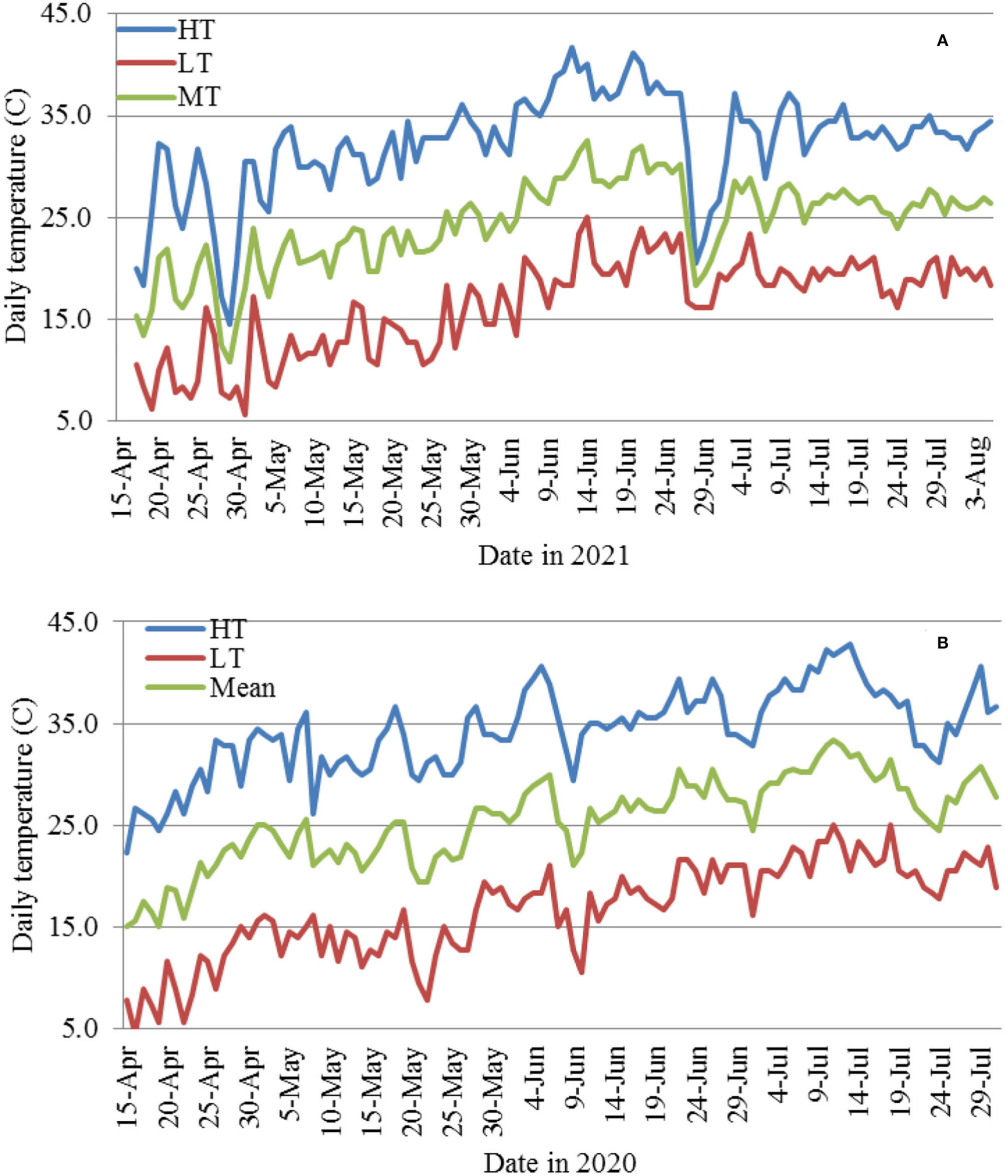
Daily high (HT), low (LT), and mean (MT) temperatures from April 15 to late July or early Aug. in 2021 **(A)** and 2020 **(B)**.

**Figure 10:**
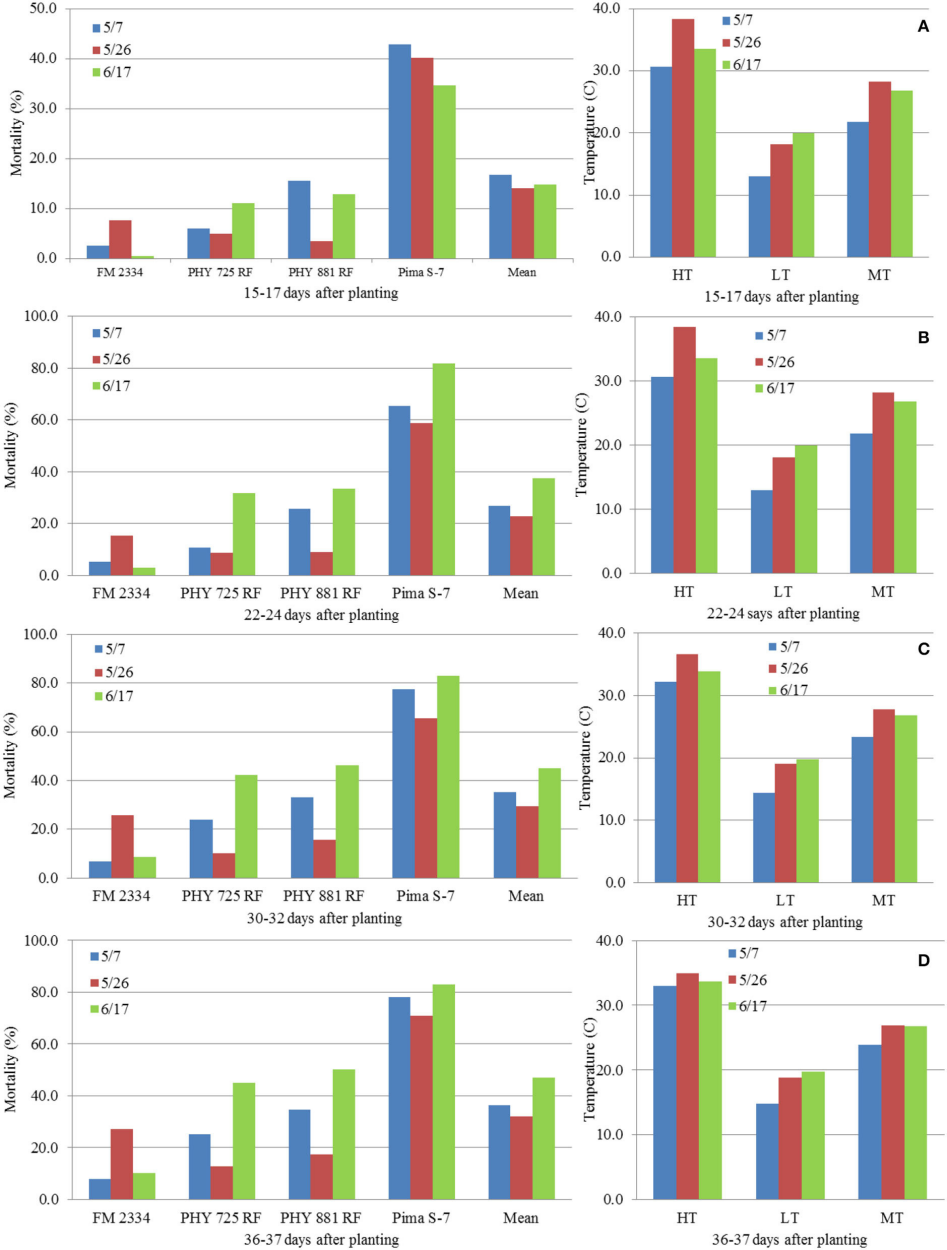
Average mortality (%) in four cotton cultivars at different days [**(A)** 15–17 DAP; **(B)** 22–24 DAP; **(C)** 30–32 DAP; **(D)** 36–37 DAP] after planting on May 5, May 26, and June 17, 2021, grown in pre-infested soil supplemented with artificial inoculation of *Fusarium oxysporum* f. sp. *vasinfectum* race 4, and average high (HT), low (LT), and mean (MT) temperatures from day of planting to the day of evaluation.

Correlation analyses were performed using data from the three planting dates in 2021 (5/7 and 5/26, and 6/17) with the exclusion of the 4/16 planting due to the lack of data before 36 DAP. MR had a consistent negative correlation with daily high temperature (HT) at the four evaluation dates (15–17, 22–24, 30–32, and 36–37 DAP) within each cultivar (except for FM 2334GLT) and on average (Table 2), although most of the correlations were not significant. Similar results were observed between MR and HT at three DAP intervals (between 15–17 and 22–24 DAP, between 22–24 and 30–32 DAP, and between 30–32 and 36–37 DAP) (Table 2). However, the relationships of MR with daily low temperature (LT) and mean temperature (MT) were inconsistent (from negative to positive). For 16 days between June 10 and 26 (Figure 9), the daily HT ranged from 37.2 to 41.1°C, while the LT ranged between 18.3 and 23.9°C which were still favorable for FOV4. The plants from seeds planted on 5/7 were too old to be affected, while the 6/17 planting was still at the emergence stage and was therefore too early to be affected (in effect, the high temperature encouraged seed germination and suppressed FOV4 infection). However, seedlings from the 5/26 planting were at the 1–2 true leaf stage and symptom development including mortality was suppressed by the HT during this period. This high-temperature period was followed by a period of lower temperature (with daily HT of 20.6–34.4°C except for 3 July with 37.2°C) between 27 June (10 DAP) and 8 July (21 DAP) (Figure 9A), which favored FOV4 infections in seedlings of the 6/17 planting. Therefore, the 5/26 planting had the lowest MR, followed by the 5/7 planting among the three planting dates at all the four DAP except for 15–17 DAP for the four cultivars except for FM 2334GLT which had higher MR at the 5/26 planting date (Figure 10). It appeared that daily LT and MT did not affect MR, but MR in the moderately resistant FM 2334GLT was not affected by HT. It suggested that MR in FM 2334GLT may not be affected by temperature. Planting dates had less effects on MR in highly susceptible Pima S-7 and moderately resistant FM 2334GLT, but greater effects on moderately susceptible PHY 725 RF and moderately resistant Pima PHY 881 RF.

**Table 2:** Coefficients of correlation (*r*) between mortality caused by Fusarium wilt race 4 (*Fusarium oxysporum* f. sp. *vasinfectum*) and average daily high (HT), low (LT), and mean (MT) temperatures (Temp) at different days after planting (DAP) from three planting dates (5/7, 5/26, and 6/17) in 2021.

**DAP**	**Temp**	**FM 2334GLT**	**PHY 725 RF**	**PHY 881 RF**	**Pima S-7**	**Mean**	Average r*
15–17	HT	0.787	−0.299	−0.987*	−0.192	−0.920	−0.493
	LT	−0.027	0.588	−0.460	−0.903	−0.863	−0.258
	MT	0.440	0.146	−0.819	−0.600	−0.999	−0.424
22–23	HT	0.791	−0.128	−0.702	−0.322	−0.300	−0.384
	LT	0.087	0.641	0.046	0.476	0.497	0.388
	MT	0.395	0.368	−0.269	0.177	0.200	0.092
30–32	HT	0.939	−0.994**	−0.513	−0.089	−0.939	−0.532
	LT	0.994**	−0.941	−0.082	−0.519	−0.994**	−0.514
	MT	0.992**	−0.994**	−0.309	−0.309	−0.992**	−0.537
36–37	HT	0.963*	−0.517	−0.650	−0.705	−0.429	−0.624
	LT	0.437	0.299	0.139	0.065	0.392	0.168
	MT	0.621	0.086	−0.078	−0.152	0.185	−0.048
Interval 16–23 DAP							
	HT	0.999**	−0.541	−0.728	−0.594	−0.555	−0.621
	LT	0.453	0.458	0.229	0.399	0.443	0.362
	MT	0.787	0.040	−0.203	−0.026	0.023	−0.063
23–31 DAP	HT	0.939	−0.994**	−0.513	−0.089	−0.939	−0.532
	LT	0.994**	−0.941	−0.082	−0.519	−0.994**	−0.514
	MT	0.992**	−0.994**	−0.309	−0.309	−0.992**	−0.537
31–37 DAP	HT	−0.503	−0.730	−0.102	−0.786	−0.999**	−0.539
	LT	0.976*	0.872	0.979*	−0.351	0.345	0.500
	MT	−0.355	−0.567	−0.239	−0.324	−0.273	−0.377

r 0.10 = 0.951; r 0.05 = 0.988; r 0.01 = 1.000; ^*^Excluding FM 2334GLT.

**Figure 11:**
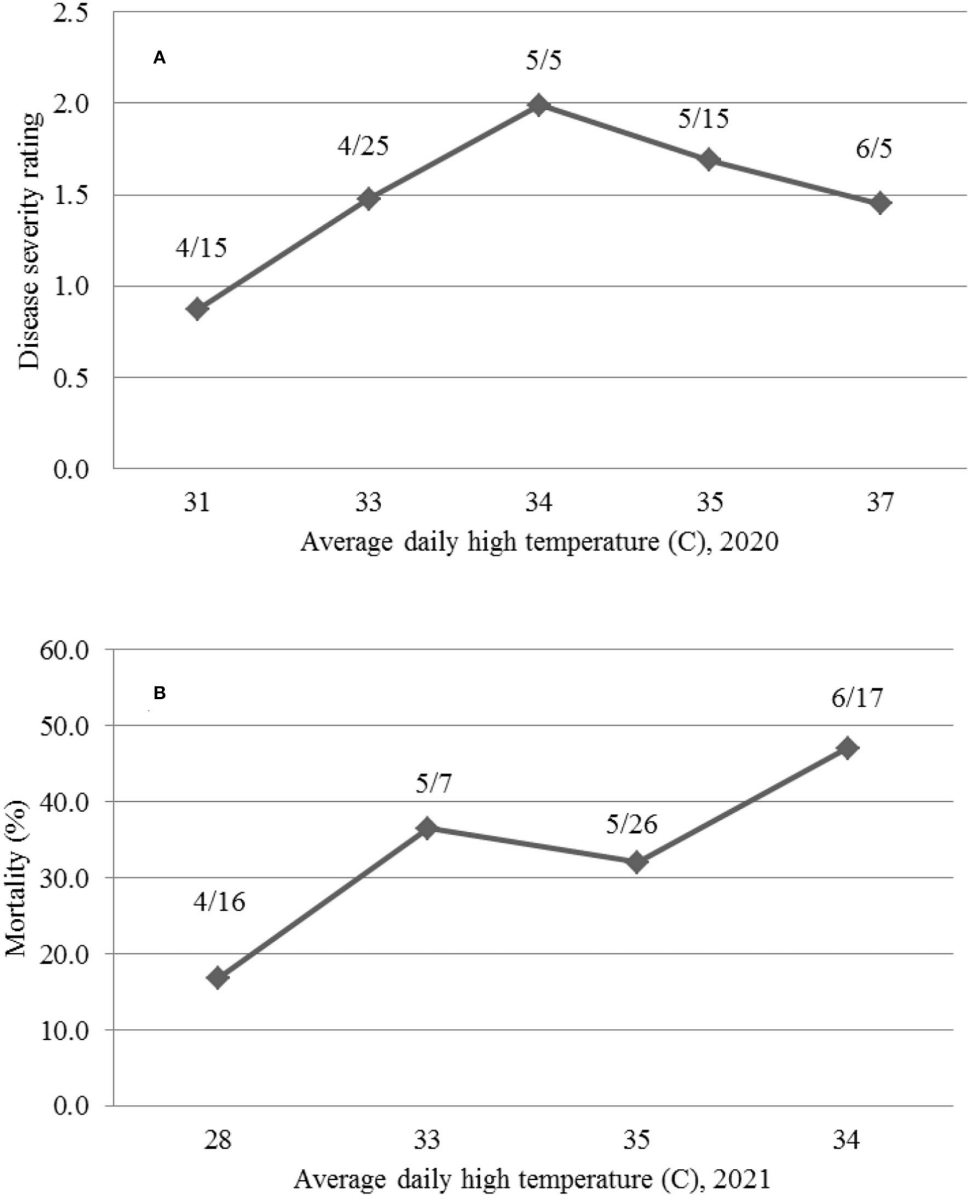
The relationship between average daily temperature (C) and the mean disease severity rating **(A)** caused by *Fusarium oxysporum* f. sp. *vasinfectum* race 4 at 30 days post-inoculation (DPI) in 2020 and mean mortality **(B)** caused by *Fusarium oxysporum* f. sp. *vasinfectum* race 4 at 36 days after planting (DAP) in 2021 for different planting dates (month/day).

The results in 2021 suggest that daily high temperatures at or above 35°C suppress MR. The 2020 planting results also support the above observations in that the overall daily HT for the 4/15 and 4/25 and 5/5 plantings were 30.9, 33.0, and 33.6°C, respectively; and the DSR for the three planting dates increased from 0.87 to 1.99. However, when the average daily HT reached 34.7 and 37.1°C for the 5/15 and 6/5 planting dates, the DSR decreased to 1.69 and 1.45, respectively. The higher DSR at the 5/5 planting date was likely due to two periods of low HT (29.4–31.7°C from May 20 to 26 at the 1-leaf stage and 5 days of 29.4–34.4°C) between June 8 and 16 (Figure 9B). The relationship between the average daily HT after planting and DSR or MR in the two years can be seen in Figure 11. In both years, DSR or MR increased from average daily HT at 28°C to that at 34°C, then decreased at 35°C and further decreased at 37°C.

## Discussion

### Planting Dates, FOV4 Disease Severity, and Temperature

As seen above, none of the planting dates in 2020 including early in the growing season (mid-April to early May) induced high MR as observed under field or low-temperature conditions (Zhang et al., 2020a). We speculated that the growth stage of cotton may have played a role in that inoculation after the 2-leaf stage caused low MR (as supported by another study, Zhu et al., unpublished). Therefore, to mimic field conditions, we used a FOV4 pre-infested soil for planting seeds in 2021, followed by an artificial inoculation on each planting date (16 April, 7 and 26 May, and 16 June) with a 19–21 day interval between two neighboring planting dates. Another change made was to use 19-L pots instead of 10-cm pots (0.8 L in size) to allow more seeds (50) to be planted in each pot. We also added a highly susceptible cultivar, Pima S-7, and replaced Pima PHY 841 RF with Pima PHY 881 RF because of seed availability (but both had similar pedigrees and the same resistance source for FOV4 from Pima S-6).

Fusarium wilt by FOV4 is an early season soil-borne fungal disease, causing plant mortality from seedling emergence until the square stage. In this 2-year study of planting dates between mid-April and early or mid-June, we showed that delay in planting did not decrease disease severity caused by FOV4. In fact, the usual local mid-April planting had the lowest disease severity (DSR or MR) in both 2020 and 2021, which was increased at the late April and early May plantings. In 2020, DSR reached the highest at the 5/5 planting, which was followed by a gradual reduction in the 5/15 and 6/5 plantings. In 2021, MR was also followed by a decrease in the late May planting, but it reached the highest in the 6/17 planting. However, for the mid-April planting, it was likely that some seeds might not germinate (due to seed rot) because of low soil temperature and/or FOV4 infections, which could underestimate DSR or MR. This was why this planting date was not included in comparing DAP and MR among four different planting dates in 2021. Therefore, seed germination, seed rot, and seedling emergence should be studied under FOV4-infested field conditions at different planting dates in the future.

We further showed that differences in DSR or MR among planting dates were not associated with LT, because daily LT among the planting dates between mid-April and mid-June in the desert southwest US are favorable for FOV4 infections. However, the results in both 2020 and 2021 support the conclusion that HT at or above 35°C suppresses FOV4 disease development. In 2020, DSR was the highest for plants from the 5/5 planting date when the average daily HT between the planting date and when the plants were evaluated at 30 DPI was 33.6°C. However, DSR was decreased when the average daily HT increased to 34.7°C (the 5/15 planting) and further decreased when the daily HT averaged 37.1°C (the 6/5 planting). In 2021, at 36 DAP, MR increased from 16.9% at the 4/16 planting date to 36.5% at the 5/7 planting date when the average daily HT increased from 28.1 to 33.0°C. However, MR decreased to 32.0% at the 5/26 planting date when the daily HT averaged 34.9°C and then increased to the highest (47.1%) at the 6/17 planting date when HT averaged 33.7°C. It should be mentioned that the temperature data used in this study were from a local weather station, not actual soil temperatures. Soil temperatures are usually lower in the summer and fall and higher in the spring and winter, but close correlations between air temperatures and soil temperatures at different depths have often been observed (Zhan et al., 2019). Infection and disease development by FOV4 are affected by both soil (early root infection in the soil) and air temperature (disease development through the spread of FOV4 to the above ground). There is an indication that favorable temperatures for root infection and disease development may differ (Ebbels, 1975). Since this study was pot-based, both soil and air temperatures were similar. In our previous greenhouse studies (Zhang et al., 2020a), we speculated that soil and air temperature would likely be 32°C or even higher on average to suppress FOV4-associated disease severity including seedling mortality. In a follow-up growth chamber study with constant temperature settings, Zhang et al. (2021a) showed that 23°C caused the highest DSR and MR in cotton, followed by 20 and 26°C, and 29°C in descending order, due to the highest mycelial growth rate at 23/26°C. In a glasshouse study, Wang et al. (1999) demonstrated that 28–33°C suppressed the symptom development on Upland cotton induced by an Australian FOV strain, while the highest disease severity occurred at 18–23°C after inoculation. However, in an early study (Young, 1923), soil temperature as high as 30.5°C was found to be optimal for wilt disease development in Upland cotton caused by FOV, but little wilt was developed below this temperature. Therefore, the mean temperature under natural conditions with fluctuations in daily temperatures and among days has different effects on FOV4 infection and disease development in cotton from the same mean temperature in a growth chamber but with a constant temperature setting.

### Inoculum Density and Disease Development

In this study, different inoculum densities were compared for their effects on disease development. However, very low MR was observed due to artificial inoculation at the 2-true leaf stage. A linear relationship between inoculum density and DSR was still detected when FOV4 conidia were inoculated to non-infested soil when seedlings were at the 2-true leaf stage. The results were somewhat different from Hao et al. (2009) who showed that DSR increased from inoculum levels of 10^3^ conidia g^−1^ of soil to 10^5^ at which it reached the highest (with no increase at 10^6^) in the highly susceptible cultivar Pima DP 744. However, in the moderately susceptible Upland Ultima, DSR continued to increase at the inoculum level of 10^6^ conidia g^−1^ of soil and no higher FOV4 spore concentration was used. Therefore, the optimal inoculum density may depend on the susceptibility level of a cotton cultivar. In our study, Pima S-7 was highly susceptible to FOV4, similar to Pima DP 744; and other cultivars (FM 2334GLT, PHY 725 RF, Pima PHY 841 RF, or Pima PHY 881 RF) were moderately susceptible or moderately resistant. In addition, FOV4 inoculum density may have interactions with different temperatures and growth stages of cotton when an artificial inoculation is made, which may affect the relationship between inoculum density and disease development. Planting seeds in FOV4-infested soil induces disease earlier, faster, and more severe than inoculation at the 2-true leaf stage. It should be recognized that a potting soil mix is different from farm soils with different soil textures, organic matter, and microbes. Inoculum density by soil weight varies in soil types with the same volume. Therefore, spore counts by soil volume may be more useful than by soil weight. In artificial inoculations, the inoculum density of 10^6^ spores on a plant basis is often recommended and used regardless of soil type.

### Cultivar Differences in Disease Progression

The results between the 2 years could not be combined in analysis or directly compared due to the use of different planting and inoculation methods. Although MR was low in 2020, due to artificial inoculation at the 2-true leaf stage, the overall results for the three cultivars as reflected by DSR were consistent with those based on MR in 2021 when FOV4-infested soils with the supplement of artificial inoculation were used. The addition of a highly susceptible Pima S-7 in 2021 was successful in separating its response from the other three cultivars. The differential responses to FOV4 among the three or four cultivars were consistent with our previous results (Zhang et al., 2021a; Zhu et al., 2021a).

Due to different responses to FOV4 among different cotton cultivars, the disease progression caused by FOV4 may follow a sigmoid model if some of the cultivars have resistance genes in response to FOV4 infections during early stages of infections at both 7 and 14 days post-inoculations, as shown by Zhang et al. (2021b). It is also a well-known fact that many genes or quantitative trait loci (QTLs) confer adult resistance but not at the seedling stage in cereal crops (Chen, 2013; Zhou et al., 2019; Yuan et al., 2020). Our results showed that the increase in mortality for highly susceptible genotypes like the Pima S-7 cultivar (with ca. 90% MR) would slow as mortality approached the maximum value, while for less susceptible cultivars, mortality continued at a linear rate for all three planting dates (5/7, 5/26, and 6/17). The experimental setup with pots, as opposed to a field setting, did not allow data collection of these more resistant cultivars to a point where mortality reached a maximum value. FM 2334GLT, PHY 725 RF, and Pima PHY 881 RF had MR ranging between 20 and 60%, suggesting various percentages of resistant genotypes, as shown in our previous greenhouse and field studies (Zhang et al., 2020a,c). For cultivars possessing certain proportions of resistant genotypes or genotypes with different levels of resistance possessing major or minor disease resistance genes (without immune response), disease progression may be linear or sigmoid (Zhang et al., 2021b). The FOV4 resistance in Pima PHY 881 RF and Pima PHY 841 RF is known to derive from Pima PHY 800 which is derived from resistant Pima S-6, and Pima S-6 possesses a major resistance gene to FOV4 (Ulloa et al., 2013). However, these resistant cultivars are not homozygous in resistance as reflected by their various levels of MR. Many QTLs for resistance to FOV4 in Upland cotton have been recently reported (Zhang et al., 2015, 2022; Wang et al., 2018; Abdelraheem et al., 2020). Our studies show that the disease progression model is plant genotype dependent. However, regardless of genotypes, disease progression has a linear relationship with time during the early stage infection (up to 30 DAP for FOV4). In this study, highly susceptible Pima S-7 had the highest regression coefficients on each planting date, while FM 2334 RF had the lowest regression coefficients on two planting dates (5/7 and 6/17), and PHY 725 RF and Pima PHY 881 RF had similar and intermediate regression coefficients. Thus, the regression coefficient (slope) can be used to compare levels of resistance among different cotton germplasm lines instead of using DSR or MR at only one-time point, in addition to the area under the disease progress curve (AUDPC) that is used to quantitatively summarize disease severity over time, for comparison among cultivars (Simko and Piepho, 2012; Fernández-Campos et al., 2020; Serumaga et al., 2020; Zhu et al., 2021a,b). Fernández-Campos et al. (2020) recently showed that logistic models (polycyclic) provided the best fitting disease progress curve in all eight tested wheat cultivars against the wheat blast disease, which was followed by the Gompertz model; however, epidemiological parameters differed between genotypes. Therefore, epidemiological criteria can be used in breeding for line selection or cultivar releases.

In addition, temperature and plant growth stages may affect disease development and therefore disease progression models. Based on Jeffers and Roberts (1993), disease progression (mortality) in field-grown cotton caused by FOV (likely race 1) followed a linear or polynomial relationship with growing degree days (>12°C), depending on planting date and cultivar. Therefore, there is a need to perform similar studies on FOV4 using different cultivars with different planting dates under field conditions.

## Data Availability Statement

The original contributions presented in the study are included in the article/supplementary materials, further inquiries can be directed to the corresponding authors.

## Author Contributions

JZ conducted the study and drafted the manuscript. AA participated in the study and performed part of the data analysis. YZ and HE-A participated in the study. JD and TWh participated in the study and edited the manuscript. KH and TWe supported the study and edited the manuscript. TWe performed part of the data analysis and contributed to the writing of the manuscript. All authors contributed to the article and approved the submitted version.

## Funding

This study was in part supported by Cotton Incorporated, USDA-ARS, Texas A&M Agrilife Research, and New Mexico Agricultural Experiment Station.

## Conflict of Interest

KH and TWe were employed by Cotton Incorporated. The remaining authors declare that the research was conducted in the absence of any commercial or financial relationships that could be construed as a potential conflict of interest.

## Publisher's Note

All claims expressed in this article are solely those of the authors and do not necessarily represent those of their affiliated organizations, or those of the publisher, the editors and the reviewers. Any product that may be evaluated in this article, or claim that may be made by its manufacturer, is not guaranteed or endorsed by the publisher.
